# The Reduced Predictive Value of Interleukin 28b Gene Polymorphisms in a Cohort of Patients With Thyroiditis Developed During Antiviral Therapy for Chronic Hepatitis C: A Preliminary Study

**DOI:** 10.5812/hepatmon.6036

**Published:** 2012-08-30

**Authors:** Huy A Tran, Tracey L Jones, Elizabeth A Ianna, Robert A Gibson, Glenn E M Reeves

**Affiliations:** 1 Hunter Area Pathology Service, John Hunter Hospital, Newcastle, Australia; 2 Hepatitis C Service, Gastroenterology Department, John Hunter Hospital, Newcastle, Australia

**Keywords:** Polymorphism, Genetic, Hepatitis C, Thyroiditis

## Abstract

**Background:**

Single nucleotide polymorphism in the interleukin28B (IL28B) gene was recently shown to be associated with a significant increase in response to interferon-α and ribavirin treatment in patients with chronic hepatitis C. Similarly, thyroid disease (TD) occurring during treatment confer an improved sustained virologic response (SVR).

**Objectives:**

To determine the role of IL28B genotypes in a cohort of hepatitis C patients who develop TD during treatment and its relationship to SVR.

**Patients and Methods:**

IL28B gene profiles including rs12979860, rs12980275 and rs 8099917 and their genotypes were determined in a cohort of 23 hepatitis C patients who developed TD during treatment and their relationship to SVR.

**Results:**

Out of 23 studies cases, 19 has one or more favorable genotypes, of which 15 (78.9%) achieved SVR. Eleven has all three unfavorable genotypes and yet achieved 72.7 % SVR. The presence of more than one favorable genotype only correctly predicts SVR vs. non- SVR in ~50 % of cases, i.e. by chance.

**Conclusions:**

Despite the small number of subjects, the presence of one or more unfavorable IL28B genotype does not portend a poor SVR prognostic outcome. This suggests that TD in this clinical context may be a critical factor in the achievement of SVR, probably above that of the genetic predisposition.

## 1. Background

It was discovered in 2009 that a single nucleotide polymorphism (SNP), rs12980275, three kb upstream of the IL28B gene confers more than a twofold response to combination interferon-α (IFN-α) and ribavirin (RBV) treatment (1). This is also applicable to the SNP rs8099917 and rs12979860 (2, 3). In addition, it was observed that this genotype also strongly enhances the spontaneous and natural resolution of acute HCV infection, especially in Europeans and Africans (4). Furthermore, it has been observed that patients who developed thyroid diseases (TD) during treatment with IFN therapy also have a six fold increase in achieving sustained virologic response (SVR) (5). A plausible reconciliation of these observations is that these TD patients harbor the aforementioned favorable SNPs which may explain the extra advantageous virologic response. If proven, it may possibly lead to potential future advances in the treatment of hepatitis C with thyroid hormones, albeit adjunctive.

## 2. Objectives

This study therefore aimed to explore this potential and important linkage and to determine the predictive values of these genetic parameters. The aim is to determine the frequency of SNPs rs12979860, rs12980275 and rs8099917 and their genotype in patients who develop TD during combination IFN-α and RBV therapy. These are then correlated to the final SVR to see if the favorable SNP and/or specific genotype profiles are able to predict the favorable outcome.

## 3. Patients and Methods

### 3.1. Patients

The patients were recruited from a Hepatitis C service center in a major tertiary referral hospital. In total 23 patients were available. All consented to the study. Ethical approval was not required as the study was clinically indicated. All were medication naive, i.e. all were undergoing therapy for the first time. All other causes of chronic hepatitis were excluded including hepatitis B and chronic alcoholic liver disease.

### 3.2. Laboratory Assay Characteristics

IL28B assay was performed as previously described (2). Three SNPs were performed on each patient, namely: rs12979860, rs12980275 and rs8099917 and their alleles. Most samples were performed after treatment had been completed as the SNP discovery was not made till the end of 2009. Four cases had their samples tested during the course of treatment.

### 3.3. Laboratory Thyroid Assay Characteristics

Third generation serum thyrotropin (TSH) and serum fT4 levels were determined by two-site sandwich immunoassay using an automated chemiluminescent system (Diagnostic Products Corporation, Immulite 2000). The reference range (RR) for TSH was 0.4 – 4.0 mU/L and fT4 10.0–26.0pmol/L. The coefficients of variation (CV) were 5.0 % and 5.1 % at TSH concentrations of 4.0 mU/L and 10.0 mU/L respectively. For fT4, the CV was 6.5 % at 10.0 pmol/L. Similarly, fT3 levels were performed using a two-site sandwich immunoassay using an automated chemiluminescent system (Beckman Coulter DXI). The RR was 3.5 – 6.0 pmol/L with 8.7 % CV at 6.0 pmol/L.

### 3.4. Statistical Analysis

Statistical analyses were performed using Stata version 10 (Stata Corporation, College Station, Texas, USA). Clinical and measured variables were summarized as means ± standard deviations (with 95 % Confidence Intervals) for continuous data, and as proportions (with percentages) for categorical information. Differences between these summary statistics were analyzed using the Student t and Mann–Whitney–Wilcoxon tests for continuous variables and the χ^2^ test for categorical variables. The Fisher exact test was employed when the minimum expected value with χ^2^ testing was less than five. Linear regression analysis was used to correlate measured parameters. Correlation coefficients and significance levels were calculated using standard methods. A probability value of P < 0.05 (two-sided), except where otherwise specified, was considered significant. Exploratory analysis of all variables was performed using pair-wise correlation analysis and t–testing with nominal logistic regression models to be fitted using dichotomous outcome variables. Only variables displaying significant values on t–testing or logistic regression were to be regarded as clinically useful. Correlation comparison was performed between individual favorable SNP genotypes and final SVR. Each genotype variant is assigned a likelihood of response according to the initial population studies (1, 2, 3, 4), Table 1. A likelihood of response of > 50.0 % from individual genotype was arbitrarily deemed favorable and vice versa. The most favorable genotypes are: CC for rs12979860, AA for rs12980275 and TT for rs8099917. A multivariate analysis was performed to analyze all variables in Table 1 in relation to SVR.

**Table 1:** The Likelihoods of Responses of Each Individual Single Nucleotide Polymorphism (SNP) Genotypes in Percentages, Some Parts are Diverted From Studies by Tanaka Y, et al., Suppiah V, et al., Thomas DL, et al. and Ge D, et al. respectively (1, 2, 3, 4). The Favorable Genotypes for Each Individual SNP are Underlined and Unfavorable are Normal.

**Genotypes **	** Likelihood of Response, % **
** RS 12979860 **	
CC	80
CT	40
TT	32
** RS 12980275 **	
AA	61
AG	41
GG	32
**RS 8099917 **	
TT	56
GT	36
GG	32

## 4. Results

Baseline characteristics of all 23 studied subjects are presented in Table 2 including the thyroid diagnoses and SNP genotypes. In this cohort, the most prevalent genotypes for rs12979860, rs12980275 and rs8099917 are CT (60.9 %), AG (56.5 %) and TT (52.2 %) respectively. Table 3 individualizes the various SNP genotype frequencies in our cohort and their relationships to SVR in all 23 subjects. These are compared with data from the literature (4, 6, 7) for the same genotypes in the HCV population. For rs12979860, genotype CC is least prevalent in the TD group, 17.4 % vs. 38.3 % while genotype TT appears similar although the data is inconsistent, 21.7 % vs. 13.6 % and 25.4 %. For rs12980275, AA is least frequent at 30.4 % vs. 40.2 %. The rest of the genotypes including SNP rs8099917 are similar in both cohorts of thyroid and non-thyroid HCV groups. Table 4 illustrates the number of individual favorable genotype in relation to SVR. As a favorable genotype is arbitrarily defined as > 50 %, this only applies to genotype CC in rs12979860, AA in rs12980275 and TT in rs8099917, Table 1. Nineteen out of 23 (82.6 %) has one be or more unfavorable genotypes of which 15 (78.9 %) achieve SVR. Eleven out of 23 (47.8 %) has all three unfavorable genotypes, yet achieving a 72.7 % SVR rate. Overall, 12 has 1, 7 has 2 and 4 has 3 favorable genotypes. A multivariate analysis indicates that the presence of more than one favorable genotype completely predicts SVR. One patient with one favorable genotype did not achieve SVR although the likelihood of response for that particular genotype (TT rs8099917) is only modest at 56 % (patient 12, Table 2). Overall, un invariant logistic regression analysis revealed no relationship between SVR and all variables displayed in Table 1. Subsequent multivariate analysis showed a similar lack of association.

**Table 2:**
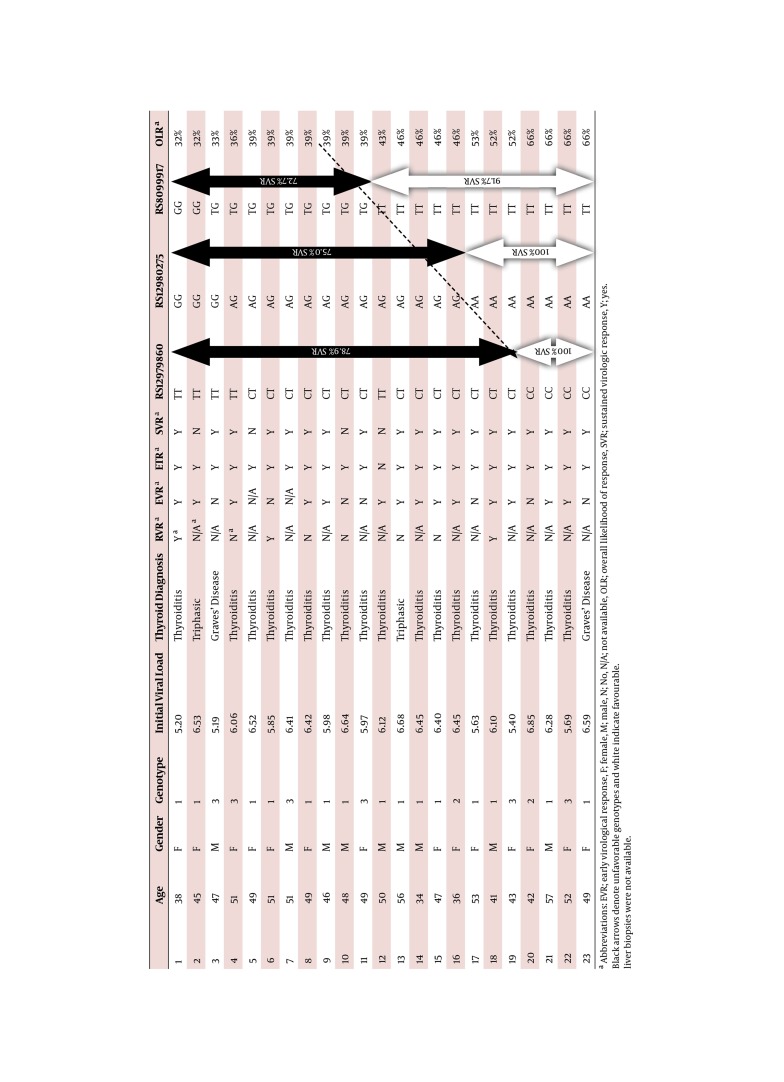
Baseline Characteristics, Hepatitis Outcomes, Thyroid Functions and SNPs Profiles 23 Patients

**Table 3:** Individual IL28B Genotype Frequencies in Our Cohort Compared With Those in the General Literature. Some parts are diverted from studies by Thomas DL, et al., Sarrazin C, et al. and Li S, et al. respectively (3, 6, 7).

	**RS 12979860, % (n = 23)**			**RS 12980275, % (n = 23)**			**RS 8099917, % (n = 23)**		
	**CC 4 (17.4)**	**CT 14 (60.9)**	**TT 5 (21.7)**	**AA 7 (30.4)**	**AG 13 (56.5)**	**GG 3 (13.1)**	**TT 12 (52.2)**	**GT 9 (39.1)**	**GG 2 (8.7)**
SVR a, No. (%)	4 (100.0)	10 (71.4)	3 (60.0)	7 (100.0)	10 (76.9)	2 (66.7)	11 (91.7)	7 (77.8)	1 (50.0)
Thomas et al. (3), %	42.5%	32.1%	25.4%	Not Applicable	Not Applicable	Not Applicable	Not Applicable	Not Applicable	Not Applicable
Kurosaki et al. (6), %	Not Applicable	Not Applicable	Not Applicable	Not Applicable	Not Applicable	Not Applicable	70	29	1
Sarrazin et al. (7), %	38.3	48.1	13.6	40.2	47.3	12.5	60.2	35.4	4.4

^a^Abbreviation: SVR, sustained virologic respons

**Table 4:** The Number of Individual Favorable IL28B Genotypes and Their Relationship to Sustained Virologic Response. Note: A Favourable Genotype is Arbitrarily Defined as Having a Likelihood Response of > 50 %.

**Patients (n = 23)**	**Favourable Genotype on IL28B Profiling, No.**				
**SVR a**					
Yes	8	4	3	4	19
No	3	1	0	0	4
**Total**	11	5	3	4	23

^a^Abbreviation: SVR, sustained virologic response

## 5. Discussion

**Figure 1:**
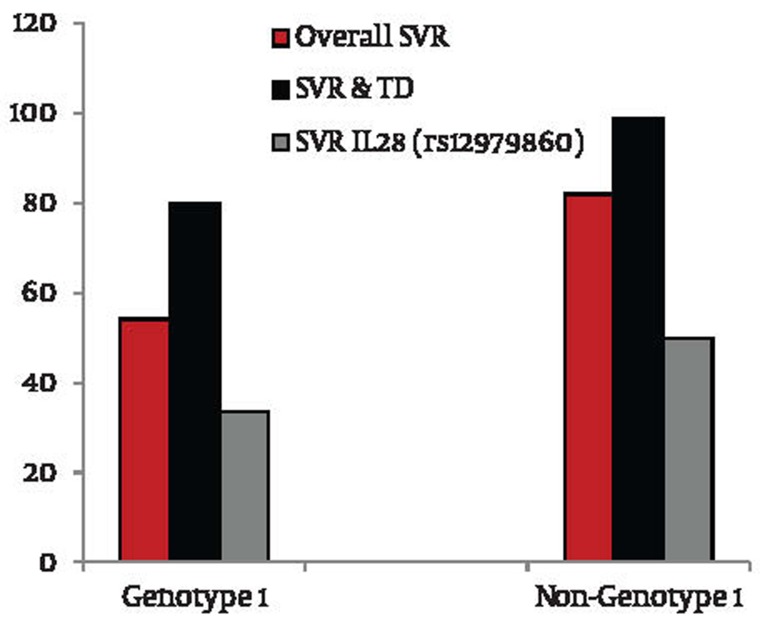
Sustained Virologic Responses Rates: Overall, With Thyroid Disease and Favorable IL28B Genotypes, Some parts are diverted from studies by Tran H, et al. ,Vilcek J, et al. and EASL Clinical Practice Guidelines respectively (5, 9, 23)

**Figure 2:**
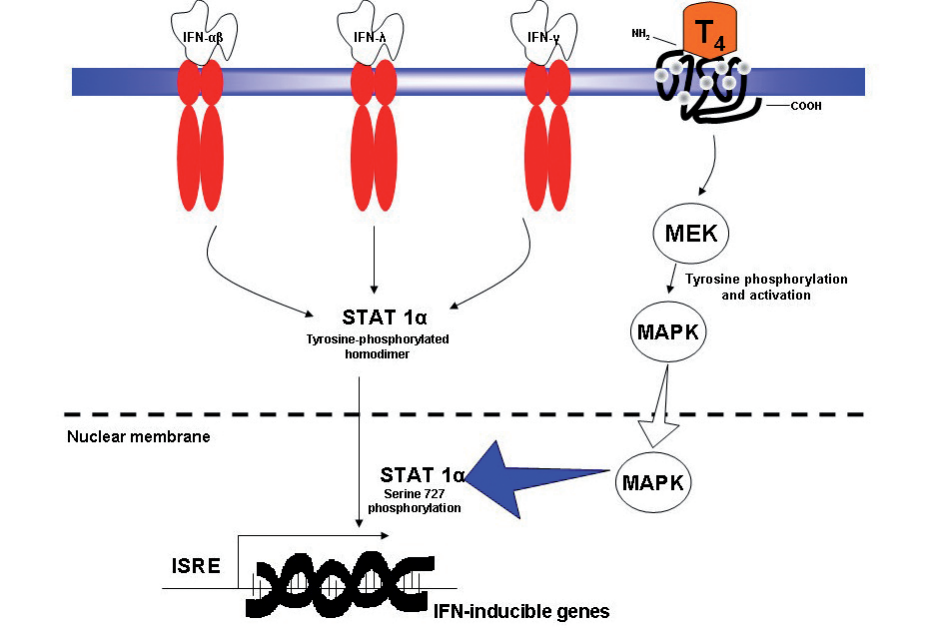
The Cellular Mechanism of the Synergy Between Thyroxin and Interferons Abbreviations: IFN: Interferon, T4: Thyroxin, STAT: Signal Transducer and Activation of Transcription, MAPK: Mitogen–Activated Protein Kinase, MEK: MAPK Kinase, ISRE: Interferon Stimulated Response Element

This is the first paper to look at the IL28B profile in this unique but small cohort of HCV patients who develop TD during treatment with combination IFN-α and RBV. This analysis was performed because one of the novel findings in recent genetic and hepatic literature is the discovery of the gene IL28B and its response to treatment in chronic hepatitis C patients (1, 2, 3). It was been shown that patients who harbor the favorable SNPs of these genes experience a favorable outcome with regards to both spontaneous clearance and response to IFN-α therapy (4). The second recent significant observation is that patients who develop TD, predominantly thyroiditis, are much more likely to achieve SVR than those who do not (5). It is therefore logical to determine if treated HCV patients who developed thyroid disease harbor the favorable IL28B variants. This may help to understand and reconcile the high SVR in patients with TD. Firstly, the commonest genotypes are heterozygotes in rs12979860 and rs12980275, CT and AG respectively where as in rs8099917 it is the homozygous TT variant. This is not dissimilar to those without TD and those in the healthy general population, Table 3. As the TT variant is the commonest favorable genotype in the thyroid group at 52.2 %, it may be the major determinant in achieving the final SVR but noting that its own likelihood of SVR is modest at 56 %. Where there is one or more favorable genotype, the development of TD ensures that SVR will be achieved in a mixture of genotypes 1, 2 and 3 although our data is early and the number is small (Table 2, patients 12 to 23). Secondly, 11 patients do not carry any favorable genotypes. Despite this, the majority achieves SVR at 72.7 % (eight out of 11). This suggested that the development of TD during treatment confers an additional advantage for SVR, despite genetic antagonism. Thirdly, a recent meta-analysis (8) reveals that the presence of IL28B (with any specific genotypes) delivered SVR rates of 34.5 % for genotype 1 and 80.9 % for genotype 2/3 respectively which does not appear to confer any response advantage, Figure 1. The etiologies for this encouraging SVR response in the TD cohort remain speculative but some of the potential considerations include the exposure to supra physiological concentration of thyroid hormones, an exaggerated immune response following IFN therapy or simple genetic predisposition. It has been proposed that the IL28B polymorphism is associated with reduced production of IL28B (1, 2), a Type III interferon (also known as interferon-λ3) bearing close relationship to the Type I interferon (α, β and δ) with similar antiviral effects but triggering signal transduction through a heterodimer consisting of IL10R2 and the unique IF-λR1 receptor chains. It is not known if any specific IL28B genotype is associated with a reduction of interferon-λ3 production and thereby may negate or suppress viral replication. Together with IL-28A and IL-29 which code for IFN-λ2 and IFN-λ1 respectively, IL28B forms a cytokine gene cluster on a chromosomal region mapped to 19q13 (9, 10). Expression of the cytokines encoded by these three genes can be induced by RNA virus infection (11). Interferon-λs are vital in the regulation of a number of viruses including herpes simplex virus and HBV. They induce blockade of HCV replication in time- and dose-dependent fashion (12). They also induce levels of IFN-stimulated genes and the antiviral efficacy of IFN-α. Interferon-λs inhibit HepG2 cell lysis after being infected with encephalo myocarditis virus. IL28B has a role in the regulation intrahepatic IFN-stimulated gene expression with consequences both for viral load and treatment response (13). They have also been shown to be virolytic (14, 15). The rs8099917 TT genotype theoretically may result in an enhanced type III IFN-λ response which involve the JAK/STAT pathway and may amplify the immunotherapeutic response. In spite of the extensive and understandable interest in the use of IL28B polymorphisms in predicting the likelihood of achieving SVR, our study found that the value of this parameter was limited in our cohort of patients. This contradicting data is consistent with the concept that, whilst being an important determinant of antiviral activity, the assessment of IL28B polymorphisms displays reduced predictive power in certain important subgroups of HCV patients, such as those in our study with treatment-related thyroiditis. In this specific cohort, out of 19 who had one or more unfavorable genotype, 15 achieved SVR at 78.9 %, those with two or more unfavorable genotype, the SVR is 75.0 % (12/16) whilst those with the complete trio of unaffordability has a SVR of 72.7 % (8/11), Table 2. Conversely the presence of one or more favorable IL28 polymorphisms displayed only 58 % sensitivity and 75 % specificity for the outcome of achieving SVR. One false positive was detected, with the TT genotype in SNP rs8099917 associated with non-SVR. Having more than one favorable allele is 100 % specific for attaining SVR, but regrettably, it results in correct classification of SVR vs. non-SVR in only ~50 % of cases. This result is not surprising because there are many other contributors to the chance of achieving SVR, including immune factors such as natural killer cell receptors, HLA class I and II loci, and polymorphisms for the genes: Chemokine Receptor 5δ32, IFNg-764G, IL10, IL12, Transforming Growth Factor b, and Tumor Necrosis Factor a (16). More importantly, previous studies have indicated the potentiation of IFN activities by thyroxin which may in turn contributes to SVR. These included IFN-α, β and γ and are thought to act via the action of intracellular Mitogen Activated Protein Kinase (MAPK) (17, 18) upon exposure to thyroxin (19). The MAPK then phosphorylates the STAT1a which in turns activate IFN-related gene translation, inducing and leading to the favorable outcome downstream, Figure 2. All 3 types of IFN share an indistinguishable signaling pathway downstream after receptor binding (9). For these specific reasons, it is strongly suggestive that TD confers an advantageous SVR outcome above that of genetic predisposition.

Whilst the cellular action of IFN is fascinating, the place of routine genotyping SNP remains undetermined (20, 21). Both TD and IL28B as potential factors affecting the hepatic outcomes are sporadic and beyond therapeutic manoeuvrement. The relevant SNPs are genetically determined and the development of TD is similarly unpredictable. The presence of thyroid autoantibodies before treatment doesn’t necessarily promote or reliably predict the development of TD. In addition, the development of TD does not require active intervention, only careful observation in the majority of case as thyroiditis is the commonest cause (22). Consequently, the clinical routine applications for these tests remain guarded. However, the dual presence of TD during treatment and a favorable IL28B variant is reassuring and indeed may potentially usurp the current log viral load reduction rules at 12 and 24 weeks for genotype 1, contrary to current recommendations (23). That is the presence of these two factors may mandate the complete 48-week course of therapy in genotype 1 patients irrespective of early virological response (EVR) status, either complete or partial. This is purely hypothetical however and the evidence for this is lacking. The major drawback of this report is the small number of subjects despite its interesting and explorative results. Clearly a large prospective randomized multicenter controlled study is required to conclusive address this observation. While IL28B gene polymorphism analysis provides significant (and often independent) prognostic information for achieving SVR during standard HCV antiviral therapy in unselected cohorts, this marker’s predictive value drops significantly in patients with TD. Indeed, the development of TD displays greater predictive power than other traditional predictors in our studies. The reduction of predictive power in this patient cohort is consistent with the suggestion that much of the favorable antiviral response seen in TD patients accounts for, and perhaps incorporates, these IL28B-gene (IFN-λ3 associated) effects. Examination of a larger selection of patients with and without TD to determine whether any differences exist in IL28B gene polymorphism analyses is clearly required.
